# Characterization of Poultry Gelatins Prepared by a Biotechnological Method for Targeted Changes at the Molecular Level

**DOI:** 10.3390/ijms25020916

**Published:** 2024-01-11

**Authors:** Aneta Prokopová, Pavel Mokrejš, Robert Gál, Jana Pavlačková, Anna Hurajová

**Affiliations:** 1 Department of Polymer Engineering, Faculty of Technology, Tomas Bata University in Zlín, 760 01 Zlín, Czech Republic; a_polastikova@utb.cz; 2 Department of Food Technology, Faculty of Technology, Tomas Bata University in Zlín, 760 01 Zlín, Czech Republic; gal@utb.cz; 3 Department of Fat, Surfactant and Cosmetics Technology, Faculty of Technology, Tomas Bata University in Zlín, 760 01 Zlín, Czech Republic; pavlackova@utb.cz; 4 Centre of Polymer Systems, University Institute, Tomas Bata University in Zlín, 760 01 Zlín, Czech Republic; anna.hurajova@centrum.cz

**Keywords:** antioxidant activity, biotechnology, functional groups, microbial population, molecular weight, gelatin

## Abstract

Chicken collagen is a promising raw material source for the production gelatins and hydrolysates. These can be prepared biotechnologically using proteolytic enzymes. By choosing the appropriate process conditions, such changes can be achieved at the molecular level of collagen, making it possible to prepare gelatins with targeted properties for advanced cosmetic, pharmaceutical, medical, or food applications. The present research aims to investigate model samples of chicken gelatins, focusing on: (i) antioxidant activity using 2,2-diphenyl-1-picrylhydrazyl (DPPH) and 2,2-azinobis-3-etylbenzotiazolin-6-sulfonic acid (ABTS); (ii) the distribution of molecular weights via gel permeation chromatography with refractometric detection (GPC-RID); (iii) functional groups and the configuration of polypeptide chains related to molecular-level properties using Fourier transform infrared spectroscopy (FTIR); (iv) the microbiological populations on sabouraud dextrose agar (SDA), plate count agar (PCA), tryptic soy agar (TSA), and violet red bile lactose (VRBL) using the matrix-assisted laser desorption ionization (MALDI) method. Antioxidant activity towards ABTS radicals was more than 80%; activity towards DPPH radicals was more than 69%. The molecular weights of all gelatin samples showed typical α-, β-, and γ-chains. FTIR analysis confirmed that chicken gelatins all contain typical vibrational regions for collagen cleavage products, Amides A and B, and Amides I, II, and III, at characteristic wavenumbers. A microbiological analysis of the prepared samples showed no undesirable bacteria that would limit advanced applications of the prepared products. Chicken gelatins represent a promising alternative to products made from standard collagen tissues of terrestrial animals.

## 1. Introduction

The properties of collagen are influenced by the origin and source from which it is obtained [1]. Collagen has three typical molecular fractions: α-, β-, and γ-chains; the α-chain consists of one polymer chain with a molecular weight of 80–125 kDa, the β-chain consists of two α-chains with a molecular weight of 160–250 kDa, and the γ-chain consists of three α-chains with a molecular weight of 240–375 kDa [1,2,3]. The individual chains are linked to each other by covalent and hydrogen bonds. The collagen fractions obtained by collagen processing are influenced by the production process, which may affect the amino acid structure (e.g., deamination of asparagine to aspartic acid or deamination of glutamine to glutamic acid) and the arrangement of the chains in the collagen itself [4,5,6]. In practice, acidic or basic processes are used, but proteolytic enzymes are also suggested. Enzyme technologies bring many advantages, notably lower processing temperatures and extraction times, and a low environmental burden [7,8,9]. Bioactive proteins with antioxidant properties produced by controlled enzymatic hydrolysis from animal tissues are used in pharmacy, cosmetics, medicine, and food [10,11,12,13]. Antioxidants are molecules capable of inhibiting or quenching reactive oxygen forms while inhibiting the oxidation of molecules that can produce free radicals [14,15]. Free radicals are the main factors that can accelerate glycation. Glycation is the process by which glucose molecules bind to various serum proteins in the body. These molecules can also cause oxidative stress, leading to other diseases [11,14,16,17].

The maximal permissible limits of microorganisms in products of animal origin are an essential parameter for their further application. The bacteria most frequently detected are *Enterobacteriaceae*, especially *Salmonella* and *Escherichia coli.* Other potential pathogens, such as *Listeria monocytogenes*, *Staphylococcus aureus*, and *Bacillus cereus*, are also investigated [18,19,20]. *Salmonella* is a group of facultatively anaerobic, gram-negative, rod-shaped bacteria found in the intestinal tract. The primary source is poultry eggs and their products; therefore, the detection of *Salmonella* is the priority for poultry products. *Salmonella enterica* subsp. *enterica* serovars cause gastrointestinal tract infections and are serious human pathogens [21]. *Escherichia coli* is a gram-negative, non-sporulating, facultatively anaerobic rod-shaped bacterium. It is present in the intestines of livestock, mainly cattle, sheep, and pigs, but can also occur in poultry. Therefore, *Escherichia coli* is also analyzed in these products, as the bacterium can cause intestinal diarrhoeal disease, which can be dangerous for the human body, causing dehydration [22,23]. *Listeria monocytogenes* is a rod-shaped, gram-positive, and non-sporulating bacterium. It occurs in livestock and in their breeding environment. The typical symptoms in humans are digestive problems [24]. *Bacillus cereus* is an endemic, facultatively aerobic, gram-positive, beta-hemolytic bacterium found in foods of plant origin. As with the previous bacterium, it mainly causes problems in the digestive tract [25]. *Staphylococcus aureus* is a gram-positive, toxin-producing, resistant bacterium found on human and animal skin and mucous membranes. The bacterium can cause inflammation and life-threatening sepsis [26].

Currently, most available gelatins are made from mammals of porcine or bovine origin. However, there is a growing interest in alternative sources of raw materials, where poultry or fish by-products represent up to 50% of the total weight. The main disadvantage of fish gelatins is their poor physico-chemical properties compared to traditional gelatins. When comparing poultry and fish gelatins, the properties of collagen are influenced by the imino acid content (proline and hydroxyproline) and molecular weight distribution, with poultry gelatins showing better viscoelastic and rheological properties [1,2,3]. Gelatin is a very versatile biopolymer, characterized by its good film-forming ability, transparency, non-toxicity, and biocompatibility, with a wide range of industrial applications, e.g., as a drug carrier [1,2,3,12,27]. It can be used not only in the food industry to produce confectionery or edible films that can extend the shelf life of fresh food, but also to produce pharmaceutical capsules to protect bioactive substances. More recently, gelatin has been used in the biomedical field to develop scaffolds for wound-healing and regeneration, to produce bioinks for 3D printers in the nutritional field, and to produce antioxidants and antimicrobial products [1,2,3,4]. Gelatin films form an effective barrier against oxygen, carbon dioxide, and volatile compounds. The barrier properties can be controlled in a suitable way (crosslinking), which makes them particularly favored for packaging applications [13,15]. For some applications, their hydrophilicity is a limiting factor. Introducing hydrophobic substances (plasticizers, lipids, fatty acids, waxes) into their structure reduces their hydrophilicity. The hydrophobic substances will promote barrier and antioxidant properties that affect the shelf life of the products [13,17,28,29].

Chicken collagen has similar properties to commercially available porcine or bovine collagen. It is suitable for producing pharmaceutical and biomedical materials and treating autoimmune diseases when administered orally [30]. Chao et al. confirmed the positive effects of the oral dosing of collagen peptides prepared by an enzymatic method from chicken bones on slowing skin aging by increasing the level of antioxidants in the skin [31]. The excellent solubility of chicken gelatins and hydrolysates, their good emulsifying and foaming properties, and their ability to retain water and oil predispose them to a wide range of applications as additives in the food sector. Their film-forming properties also make them suitable for the production of edible packaging and coatings. Biopolymeric fibers based on chicken collagen can be used in advanced biomedical applications to target drug delivery, immobilize enzymes, and inoculate and attach cells [32]. Poultry stomachs are an unused by-product in some countries, contributing to environmental pollution. However, this tissue contains collagen, which has high added value for the extraction of gelatins and hydrolysates [33,34]. The literature does not currently report the processing poultry stomachs into gelatins using proteolytic enzymes. However, procedures for the enzymatic cleavage of collagen from chicken skin [35], bones [36,37], livers [38], feet [29], and by-products remaining after the production of mechanically deboned chicken meat [39,40] are known.

This study builds on previously published articles by the authors [33,34,39,40,41]. The study aims to determine the antioxidant activity, molecular weight, functional groups, and microbiological population of gelatins prepared from poultry stomachs using an enzymatic method. Subsequently, the results will be compared with available products, and areas of industrial application of the prepared gelatins will be suggested.

## 2. Results and Discussion

The properties of the nine gelatins, prepared biotechnologically from chicken stomachs, are included in the study by Prokopová et al. [34].

### 2.1. Antioxidant Activity

Table 1 shows the results of scavenged DPPH and ABTS free radicals for each concentration (2, 4, 6, 8, and 10 mg/mL) of the gelatin samples. The antioxidant activity increased with an increase in solution concentration. ABTS antioxidant activity was higher compared to DPPH because ABTS scavenges hydrophilic and hydrophobic free radicals. ANOVA shows statistical differences in its antioxidant activity (compared to DPPH and ABTS) when changing the experimental conditions (Exp. No. 1–9) and tested gelatin concentrations. At a significance level of α = 0.05 (*p*-value < 0.05), with 95% confidence, there is clear statistical significance between almost all individual gelatin concentrations. For DPPH antioxidant activity, when comparing 6 mg/mL with a 10 mg/mL gelatin concentrations, and for ABTS antioxidant activity, when comparing 2 mg/mL with a 4 mg/mL gelatin concentrations, there is no statistical significance.

The literature reports deficient antioxidant activity for chicken gelatins and hydrolysates. For example, for gelatins extracted from chicken skin, the DPPH antioxidant activity was 11–17% [42]; for gelatins prepared from duck skin, this was 23% [43]. The low molecular collagen hydrolysate from chicken skin had higher DPPH antioxidant activity (22–48%) [44]. The effect of the molecular weight of gelatins on the value of the antioxidant activity was also demonstrated for fish gelatin extracted from cobia skin [45]. DPPH antioxidant activity was around 65% at a 1 mg/mL solution concentration for 10 kDa peptides, 90% for 5 kDa peptides, and 60% for 3 kDa peptides. The antioxidant DPPH activity of commercial fish gelatin increased from 13 to 24% with an increase in temperature annealing (from 120 to 160 °C) [10]. The DPPH antioxidant activity increased from 20 to 60% in fish gelatin from cobia skin with increasing gelatin concentration (4–12 mg/mL) [11]. Gelatin film prepared from tilapia skin has a % DPPH antioxidant activity of about 15%, and the ABTS antioxidant activity was about 80% [12]. Essential oils (bergamot, kaffir lime, lemon, and lime) added to the gelatin films improve the antioxidant activity [13]. In gelatin from yellowfin tuna skin (extracted with citric acid), ABTS antioxidant activity (on average, 93 ± 3%) was higher than DPPH (on average, 65 ± 15%) [14]. Gelatin from Nile tilapia extracted by the enzyme showed DPPH antioxidant activity values averaging 33% [15]. Fish gelatin from mackerel has, on average, DPPH-scavenged free radicals < 50%, compared with commercially available porcine gelatin (averaging around 70%) [16]. For fish gelatin from *Hypophthalmichthys molitrix*, the DPPH antioxidant activity was around 30% for the 0.10 mg/mL solution and around 90% for the 0.40 mg/mL solution [17].

In comparison with other chicken products (gelatins and hydrolysate) [42,43,44], in the present chicken gelatins, DPPH antioxidant activity was significantly higher (11–48% versus 79 ± 1%). The studies [10,11] found deficient antioxidant activity compared to DPPH antioxidant activity in the present study. In studies [12,13], the DPPH and ABTS antioxidant activities are significantly lower than those in the present study (89 ± 1%). Chicken gelatins have a higher antioxidant activity for DPPH and are comparable to ABTS [14]. In both studies [15,16], the antioxidant activity was significantly lower than in the chicken gelatins. Better DPPH antioxidant activity was obtained from 5 kDa gelatin peptides [45] and 0.40 mg/mL of fish gelatin [17] compared to the chicken gelatins in the present study.

Gelatins allow for the more efficient scavenging of free radicals in the presence of residual free –NH_2_ groups in their structure [14]. The significance of the observed antioxidant activity of chicken gelatins is essential to their intended applications in food and pharmacy. For practical applications, a minimum value of 60% radical scavenging activity is considered [46]. With a higher antioxidant activity of the samples, the substance binds to free radicals, especially oxygen, neutralizing their activity and protecting against oxidative stress. This mechanism extends the shelf life of food or cosmetic products due to its protective effect on the skin surface, which limits skin aging [47,48]. The high antioxidant effect of chicken gelatin can also be used in the production of soft and hard gelatin capsules to enhance the antioxidant effect of such packaging to protect encapsulated drugs, dietary supplements, or other substances [49].

### 2.2. Molecular Weight

Table 2 shows the molecular weight values for individual gelatin samples, including the polydispersity index (PDI), which expresses the distribution of molecular weights in gelatin samples. This shows a non-uniform particle size, meaning that PDI = 1 is a monodisperse system, and PDI > 1 is a polydisperse system [50,51]. The highest PDI was found for samples 3 and 6 (with a mean value of 1.05 ± 0.07), whereas the lowest PDI was in samples 1 and 7 (with a mean value of 4.50 ± 0.14). Thus, samples 3 and 6 had a higher particle size non-uniformity than samples 1 and 7. The PDI ranged from 5.1 to 9.0 for the other samples.

Table 3 shows the individual percentages of the molecular weights of α, β a γ-chains in gelatin samples; further, at molecular weights lower than α-chains and higher than γ-chains. All typical chains were in all gelatin samples. The lower collagen fractions of 0–80 kDa are the most represented in the samples, averaging 65 ± 5%. Samples 1, 2, 7, and 9 contained α-chains of >10%; in samples 3, 4, 5, 6, and 8, α-chains of <10% were found. The viscosity and gel strength partly influence the molecular weight and distribution [2]. The lowest viscosity and gel strength were in sample 7. The highest viscosity (corresponding to the highest gel strength) was found in samples 3 and 6. α-, β- and γ-chains affect the final gelatin properties; the longer the peptide chain length, the better the polypeptide network arrangement and the better the gel quality [3]. If β- or γ-chains are missing or present in small amounts in the gelatin, this is because of the hydrolysis and cleavage of peptide bonds. Samples 1, 4, 7, and 8 had lower molecular weights. These gelatin samples also had a lower gel strength and viscosity. This phenomenon may be the result of the higher degradation of α- and β-chains caused by enzymatic hydrolysis. Gelatins with a low molecular weight, due to the effect of enzymatic cleavage, are of lower quality [52,53] in contrast to gelatins 2, 3, 5, 6, and 9, with a higher gel strength, higher viscosity and higher molecular weight.

In the study by Díaz-Calderón et al., the molecular weight of the α-chain was in the interval from 35 to 120 kDa. The extraction conditions influenced the observed molecular weight. The gel strength also affects the molecular weight (the higher the gel strength, the higher the molecular weight of the chains). This fact is related to the size and diversity of the protein chain, the higher content of free hydroxyl groups (which play a role in forming the hydrogen bonds and helical structure during gel setting), the amino acid composition, and the concentration of gelatin solution. In this study, α-chains were higher, up to 160 kDa [1]. In the study [3], where gelatin from sea bass skin was extracted with CH_3_COOH, the band intensities of the α_1_- and α_2_-chains were around 120 kDa and around 220 kDa for the β-chains. The molecular weight decreased slightly with increasing extraction temperature, possibly due to the partial degradation induced by the thermal process. The molecular weights for α-chains were 124 kDa, and potential weights for β-chains were up to 260 kDa.

The study [54] focused on the extraction of gelatins from chicken feet using CH_3_COOH and determined molecular bands of around 125 kDa (α-chains) and 180 kDa (β-chains). The study also mentions ultrasound extraction, with a shift in the molecular weights of gelatins to 130 and 198 kDa, respectively. Another part of the experiment included testing commercially available bovine gelatin with band intensities for α_1_-chains around 155 kDa, for α_2_-chains around 135 kDa, and for β-chains around 195 kDa. In almost all cases, higher molecular weights occurred in the gelatins prepared from chicken stomachs according to this study. Only when compared with commercially available bovine gelatin was the molecular weight of the α-chain different (bovine gelatin 155 kDa; chicken gelatin 124 kDa).

In the study [5] focusing on the extraction of gelatins from black-bone chicken skin and feet using NaOH, the band intensities for α_1_-chains and α_2_-chains were around 135 kDa and 120 kDa for black-bone chicken feet gelatin, and around 140 kDa and 125 kDa for α_1_-chains and α_2_-chains for black-bone chicken skin. Gelatin from yellowfin tuna skin [14] treated with CH_3_COOH showed molecular weights of around 130 kDa for α_1_-chain, around 115 kDa for α_2_-chain, and around 250 kDa for β-chain. The molecular weight values of the discussed two studies [5,14] are similar to those of gelatins prepared from chicken stomachs in the present study. In the study [16] where they extracted gelatin from mackerel scad skin using CH_3_COOH, the band intensities of the α_1_- and α_2_-chains were found to be around 130 and 100 kDa, respectively, and around 200 kDa for the β-chains. Gelatin from chicken stomachs showed higher molecular weights. In the study [8] where gelatin was extracted from cobia skin and croaker skin using H_2_SO_4_, it was found that the gelatins contained α-chains (about 116 kDa), β-chains (about 215 kDa), and γ-chains (about 250 kDa). Similarly, gelatin from chicken stomachs showed higher molecular weights for both α-, β- and γ-chains; the values were 124, up to 260, and up to 400 kDa.

The molecular weight was studied using chicken feet gelatin extracted with CH_3_COOH [29], and the α_1_-, α_2_-, and β-chains were 150, 135, and 210 kDa. The study [12] focused on commercial fish gelatin from tilapia skin; the band intensities for α_1_- and α_2_-chains were around 150 kDa and 135 kDa, respectively. Gelatin from chicken stomachs showed higher molecular weights for both α- and β-chains. The values were 124 kDa and up to 260 kDa. For the gelatins extracted using 0.00–0.05% CH_3_COOH from bigeye snapper and brownstripe red snapper [55], all characteristic α_1_-, α_2_-, β-, and γ-chains were detected in the prepared gelatins, with molecular weights in the corresponding order of 116, 102, 205, and 250 kDa for the bigeye snapper, and of 120, 110, 210, and 260 kDa for the brownstripe red snapper. With increasing acid concentration during gelatin extraction, the intensity of the bands declined. Fish salmon gelatin [56] treated with CH_3_COOH showed molecular weights for the α-chain of around 120 kDa, for the β-chain of around 245 kDa, and for the γ-chain of around 375 kDa. In the study [57] aiming to compare hoki skin gelatin and commercially available bovine and porcine gelatin, molecular weights for α-chains around 100 kDa and β-chains around 190 kDa were in found samples at a concentration of 1.13 mg/mL. With a decrease in the concentrations of the solutions (0.75 and 0.38 mg/mL), the molecular weight slightly increased for all three sample types. In the studies [55,56,57], lower molecular weights were measured when compared with the present study, in which the molecular weights for α-, β- and γ-chains were found to be 124, up to 260, and up to 400 kDa, respectively. In commercial fish gelatin [10], the M_n_ was 6.6 kDa, M_w_ 26.0 kDa, and PDI 3.96. Subsequently, the fish gelatin was annealed at 120, 140, and 160 °C. The M_n_, M_w_, and PDI parameters lowered with increasing temperature. For M_n_, there was a decrease to 4.1, 1.7, and 1.4 kDa. For M_w_, there was a decrease to 12.2, 4.1, and 2.8 kDa; for PDI, there was a decrease to 2.98, 2.36, and 2.06, each time, for a given temperature. Compared with the results obtained from gelatins prepared from chicken stomachs, the values of M_n_, M_w_ (7.23, 57.96 kDa), and PDI (7.61) were higher.

### 2.3. Functional Groups

Table 4 provides numerical results regarding the peak regions for the tested gelatins, including the reference characteristic vibrational peak regions [58,59,60,61,62]. Amide A peak values are related to N–H stretching, linked via hydrogen bonding, corresponding to the primary structure of collagen.

For gelatins 1, 2, and 3, the wavenumbers were lower than those reported in the literature. This trend indicates that fewer amino groups were present in the gelatin samples, possibly related to the lower degradation that occurred during the enzymatic treatment process. Amide B peak is related to –CH_2_ asymmetrical stretching. The measured values correspond to the literature values, and it is valid that the value of the Amide B band decreased with an increase in extraction temperature; this indicates interactions between NH_3_^+^ groups and peptide chains in collagen. The C=O extension associated with CN stretching, CCN deformation, and in-plane NH bending provides the vibrational mode of Amide I. The band is typical of the helical structure of gelatin. All the measured values agree with the literature reports [58,59,60,61,62]. The inter-phase combination of CN extension and bending of the N–H peptide group determines Amide II. Compared with the literature values, the measured values are within the required interval. The Amide III band represents the extension of the amide bonds’ CN and N–H deformations. The same conclusions regarding characteristic vibrational peak regions were found in gelatin samples 4, 5, 6, 7, 8, and 9.

**Figure 1:**
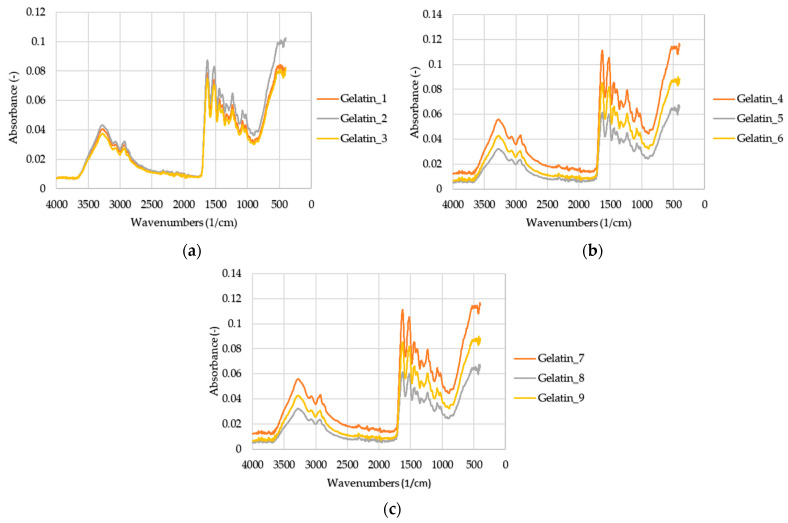
FTIR spectra of chicken gelatins: (**a**) samples 1, 2, and 3; (**b**) samples 4, 5, and 6; (**c**) samples 7, 8, and 9.

Figure 1 shows the FTIR spectra of nine gelatin samples. Figure 1a shows lower absorbance for gelatins 1 and 3 compared to gelatin 2. Figure 1b shows that the highest absorbance was found in gelatin 6, compared to gelatins 4 and 5. Figure 1c shows that the highest absorbance was found in gelatin 7, compared to gelatins 8 and 9. These phenomena will likely result from the different numbers and types of amino acids [3,7,8,10,16,28].

In the study by Jusoh, chicken skin gelatins had the characteristic peaks in Amide A, Amide I, Amide II, and Amide III at similar wavenumbers to chicken gelatins in the present study; Amide B was not found [42]. Gelatins from seabass skin showed very similar spectra. At higher extraction temperatures, the intensity of the bands shifted slightly towards higher wavenumbers [3]. In fish gelatin from mackerel, all the characteristic peaks of Amide A, Amide B, Amide I, Amide II, and Amide III were found [16]. Compared with the present study, the average peak values of Amide A were lower, while Amide B’s were higher. The study [7] focusing on the comparison of commercial porcine (PG) and bovine (BG) gelatins with sharri fish skin gelatin reports typical peaks for fish gelatin; Amide II was not found. The intensity of the sharri fish skin gelatin bands was higher when compared with both BG and PG gelatins. This difference may be explained by the different numbers and types of amino acids in the gelatin fractions due to the different extraction procedures (enzymatic or acid) that were used. In addition, fish gelatins have lower amounts of proline, hydroxyproline, and glycine; this may also affect the intensity of the observed peaks [1,2,3,7,52]. FTIR spectra were studied on commercial bovine gelatin (BG) and extracted chicken feet gelatin using CH_3_COOH; both gelatins contained all typical peaks [29]. All the peaks for chicken feet gelatin and BG were higher than those in the present study. The increased intermolecular interactions in different types of collagen can explain this phenomenon. In chicken feet gelatin, the values of vibrational peaks were lower than those in the present study; this phenomenon can be explained by the fact that the gelatin was extracted by ultrasonic treatment [54]. Chicken feet gelatin (extracted with 1.5, 3.0, and 4.5% acetic, citric, and lactic acids) contained the characteristic Amide A and B, Amide I, II, and III peaks. The average values of the FTIR spectra increased with increasing concentrations of all acids; the tested acids show a similar FTIR spectra [58]. Compared with the present study, the peak values of Amide A, Amide I, II, and III were always lower; for Amid III, identical values were observed compared to the gelatins extracted with CH_3_COOH. For Amide B, an opposite trend occurred in all cases. This difference can result from the different methods of gelatin extraction (enzymatic or acid). Gelatin from chicken feet showed typical spectral values; in comparison, in bovine commercial gelatin, Amide III was not found [59]. The values of the individual peaks, except for Amide B, were consistently lower in the present study.

### 2.4. Microbiological Population

Table 5 lists the microorganisms detected in the individual gelatin samples; bacterial values >1700 are listed here because bacterial values <1700 are not a reliable form of identification [18]. No *Salmonella*, *Listeria monocytogenes*, or *Escherichia coli* were found in any samples. In sample 4, another coliform bacterium of *Enterococcus*, *Enterococcus faecium*, was detected. *Staphylococcus aureus* was not detected in any of the gelatin samples, but bacteria from the same genus of *Staphylococcus*, *Staphylococcus hominis*, were detected in sample 5. *Bacillus cereus* was the most present bacteria in samples 1, 2, 5, 7, and 11. In addition, *Brevibacillus agri* was detected in sample 1. In sample 2, *Brevibacillus agri* and *Bacillus flexus* were detected. In sample 3, two types of *Acinetobacter* were detected: *radioresistens* and *baumannii*. In sample 4, two more bacteria, *Bacillus flexus* and *Brevibacillus agri,* were found. Sample 5 contained, in addition to *Staphylococcus hominis*, *Bacillus cereus* and *Acinetobacter baumannii*. No microorganisms with a >1700 score value were present in sample 6. *Acinetobacter radioresistens* was present in sample 7. *Acinetobacter baumannii* was found in samples 8 and 9; sample 9 also contained *Brevibacillus agri*. Samples 10, 11, and 12 were the most contaminated, with *Pseudomonas oryzihabitans*, *Bacillus flexus*, *Acinetobacter baumannii*, *Ralstonia pickettii*, *Bacillus cereus*, and *Acinetobacter radioresistens*. Yeast and fungi values were <1700 score value, and were thus not a reliable means of identification. *Enterococcus faecium* was the bacteria identified in gelatin 4 and could cause infections. Since only gelatine 4 was contaminated and *Enterococcus faecium* is present in the intestinal microflora, careless handling likely contaminated this sample. However, at a temperature of at least 70 °C for 30 min, the *Enterococcus faecium* is destroyed [63,64,65].

Evaluating gelatin microbiological characteristics is crucial to confirm if the extracted gelatin complies with the most stringent food standards [66] and pharmacopeias [67]. As part of the microbiological analysis, when colonies of bacteria with typical growth on selected growth mediums were isolated from gelatin samples, the following bacteria were detected in the gelatin samples: *Enterococcus faecium*, *Staphylococcus hominis*, *Bacillus cereus*, *Brevibacillus agri*, *Bacillus flexus*, *Acinetobacter radioresistens*, *Acinetobacter baumannii*, *Pseudomonas oryzihabitans*, and *Ralstonia pickettii*. The presence of the bacteria listed above may be due to airborne contamination, contamination from the water in which the samples were processed, or careless handling [68,69]. No representatives of the genus *Salmonella* were detected in any of the samples. Neither *Listeria monocytogenes*, *Staphylococcus aureus*, nor *Escherichia coli* were detected. The above-listed genera are absent in the compliance tests for applications of gelatins in food, pharmacy, medicine, and cosmetics [19,20,21,22,23,24,25,26]. A temperature above 70 °C inhibits all identified bacteria in the gelatin samples [70].

## 3. Materials and Methods

### 3.1. Apparatus, Tools and Chemicals

UV–VIS spectrophotometer Helios 3 Thermo Spectronic, differential refractometer Waters 2414 (Mettlet-Toledo, Ltd., Prague, Czech Republic), microjet microwave autoclave (The Rodwell Autoclave Company, London, UK), vortex mixer mini analog (OHAUS Europe GmbH, Nänikon, Switzerland), FTIR Bruker ALPHA (Bruker GmbH, Vienna, Austria), Memmert cultivator (Memmert GmbH + Co. KG, Büchenbach, Germany), Lambda Life laminar box (Lambda Life, Ltd., Bratislava, Slovakia), Waters HPLC Breeze analytical device (Waters Chromatography Europe B.V., Etten-Leur, The Netherlands), OHpak SB-804 HQ analytical column 300 × 8 mm/13µm (Altmann Analytik GmbH & Co. KG, Munich, Germany). 0.1 mol/L phosphate buffer pH 6.8 (Faren Ltd., Uherské Hradiště, Czech Republic), DPPH (2,2-diphenyl-1-picrylhydrazyl) (MedChemExpress, Monmouth Junction, NJ, USA), ABTS (2,2′-azino-bis-(3-ethylbenzothiazoline-6-sulfonic acid) (Merck Life Science, Praha, Czech Republic).

### 3.2. Samples of Extracted Gelatins

According to the study of Prokopova et al. [34], gelatins were prepared from chicken stomachs; each experiment was repeated three times. The extraction of the gelatins occurred according to the Taguchi design, a method of multifactorial experiments that allows for a statistical evaluation and exploration of the influence of independent variables (processing factors) on dependent variables, making the process less variable [71]. Taguchi’s design uses orthogonal arrays in which the design of the experiments is balanced, factor levels are weighted equally, and each factor can be assessed independently of all the other factors. Two factors at three levels were studied: factor A, the amount of enzyme added (0.10, 0.15, and 0.20%), and factor B, the extraction temperature (55.0, 62.5, and 70.0 °C). This design reduces the time needed compared to the experiment with a fractionated design. A brief description of the processing of chicken stomachs into gelatins follows. Chicken stomachs were treated with water, 0.2 mol/L NaCl, 0.06 mol/L NaOH, and a mixture of petroleum ether with ethanol to remove impurities, albumins, globulins, and fat. Purified collagen was conditioned with proteolytic enzyme Protamex^®^, a microbial endopeptidase produced by the submerged fermentation of genetically modified microorganisms, activity 1.5 AU/g (Novozymes, Bagsvaerd, Denmark) at pH 6.5 ± 0.5 at 22.0 ± 1.5 °C for 24 h to cleave a quaternary structure of collagen. The enzyme solution was filtered, and conditioned collagen was washed with cold running water. Gelatins were extracted with water (collagen water ratio 1:8) at temperatures according to factor B for 45 min. After filtration, the gelatin solutions were heated to 85.0 ± 1.0 °C for 10 min to inactivate any remaining enzyme. Finally, gelatin solutions were dried in a thin film (approx. 4 mm) at 53.0 ± 1.0 °C for 24 h. The gelatin samples were ground to a fine powder using a blender and stored in the dark in a desiccator at 20.0 ± 2.0 °C. Nine chicken gelatins were prepared; Table 6 lists the gelatin samples, along with the technological conditions of the extraction process.

### 3.3. Antioxidant Activity

The antioxidant activity of gelatin shows gelatin’s ability to remove reactive oxygen species from a sample at the specific concentration used for the analysis [11,12,13,14,15,16,17]. The study’s methodology was based on the preparation of chicken gelatin samples of different concentrations (2, 4, 6, 8, and 10 mg/mL) in distilled water; the proposed gelatin concentrations correspond to previous studies of similar samples [14,16,17,45]. The gelatins were weighed, distilled water was added, and the whole system was heated to 45.0 ± 2.0 °C to dissolve the gelatins. The preparation of the mixture solution for measurement consisted of mixing 500 µL of the gelatin sample solution with 500 µL of 95% ethanol and 125 µL of 0.2 mM DPPP or ABTS solution. The mixture was stirred in a circular motion, centrifuged at a rotational speed of 2000 rpm on a centrifuge for 3 min to settle the impurities and crystals, and then incubated in the dark at 20.0 ± 2.0 °C for 30 ± 1 min. The absorbance of the samples was measured at 517 nm on a Helios 3 Thermo Spectronic UV-VIS spectrophotometer. Calculations to determine the scavenging activities of the protein fractions towards DPPH or ABTS radicals were performed according to Equations (1) and (2):

(1) $$DPPH\;radical\;scavenging\;activity\;\left(\%\right)=\frac{AC+AB-AS}{AB}\times 100$$

(2) $$ABTS\;radical\;scavenging\;activity\;\left(\%\right)=\frac{AD+AE-AS}{AE}\times 100$$

where *AC* is the absorbance of the *DPPH* solution without gelatin samples, *AB* is the absorbance of the gelatin samples with ethanol and distilled water without DPPH, *AS* is the absorbance of the gelatin samples in distilled water, *AD* is the absorbance of the ABTS solution without the gelatin samples, and *AE* is the absorbance of the gelatin samples with ethanol and distilled water without *ABTS*.

### 3.4. Molecular Weight Distribution

The molecular weight distributions of gelatin samples were determined by gel permeation chromatography with refractometric detection (GPC-RID), and the individual values were compared with the available literature [2,3,50,51,52,53]. The molecular weights for gelatins are assumed to contain at least one α-chain and a β-chain of about 110–120 kDa and 190–210 kDa, respectively. The analytical method consisted of weighing a 2.00 ± 0.01 mg powdered sample and dissolving it in 1 mL of 0.1 mol/L phosphate buffer in a sealed vial at 20.0 ± 2.0 °C for 4 h. The Waters HPLC Breeze analytical apparatus was injected with 100 µL of the sample, the measurement process was carried out at 40.0 ± 1.0 °C, and the solution flow rate was 1 mL/min. The system was calibrated using pullulan standards in the 667–344,000 Da range. Table 7 summarizes the determined molecular weights, including the polydispersity index (PDI), calculated according to Equation (3):

(3) $$x=\frac{M_{w}}{M_{n}}$$

### 3.5. Functional Groups

The Fourier transform infrared spectroscopy (FTIR) analyzed the peak regions (Amides A and B, and Amides I, II, and III) for each gelatin sample, and the results were compared with the references [58,59,60,61,62]. The gelatins are expected to contain the typical peaks in Amides A and B, and Amides I, II, and III. The measurements were performed using the ATR method, with the platinum crystal oriented on the side facing the lamp during photo exposure. The background without gelatin was used as a control sample. The samples were exposed to infrared light ranging from 400 to 4000 1/cm. Thirty-two scans at 20.0 ± 2.0 °C were taken during one measurement. Table 8 shows the characteristic FTIR peak vibrational regions for powdered gelatins.

### 3.6. Microbiological Population

Microbiological tests identified the microorganisms in the gelatins; the methodology is based on the study by Gal et al. [18] with partial modifications. Growth mediums were prepared according to the manufacturer’s instructions (Merck KGaA, Darmstadt, Germany). A gelatin sample of 5.00 ± 0.01 g was hydrated in 45.0 ± 0.5 mL of distilled water at 37.0 ± 1.0 °C. The sample prepared in this way was inoculated on a growth medium at an amount of 100 µL. The cultivation was performed on SDA at 25.0 ± 0.5 °C for seven days, PCA samples were cultivated at 30.0 ± 0.5 °C for 24 h, and TSA and VRBL samples were cultivated at 37.0 ± 0.5 °C for 24 h. Under the above conditions, colonies grown on medium were repeatedly aseptically inoculated and cultivated on growth medium PCA, SDA, TSA, and VRBL. Samples were prepared for analysis on the MALDI-TOF Matrix-Assisted Laser by mixing the bacterial culture with 300 µL of sterile distilled water and 900 µL of 96% ethanol. Subsequently, the samples were centrifuged for 2 min at 14,000 rpm. After centrifugation, the supernatant was removed, and the pellets were centrifuged again. The supernatant was removed, and the pellets were dried and dissolved in 30 µL of 70% formic acid, and 30 µL of acetonitrile. The suspension was centrifuged at 14,000 rpm for 2 min, and 1 µL of the supernatant was applied to a MALDI plate. After drying, each sample was recoated with 1 µL 2-cyano-3-(4-hydroxyphenyl) acrylic acid and dried again. The resulting samples were isolated by nitrogen laser. The mass spectra were generated automatically using a Microflex LT MALDI-TOF (Bruker GmbH, Vienna, Austria) mass spectrometer operating in a linear positive mode in the mass range from 2 to 20 kDa. Before the actual measurement, the apparatus was calibrated using the Bruker bacterial test standard. Table 9 shows the identification criteria used to evaluate the obtained data.

### 3.7. Statistical Analysis

For antioxidant activity, a one-way ANOVA was performed at a 95% significance level (*p* < 0.05) using Microsoft Office Excel 2016 (Denver, CO, USA). For molecular weight, the data were processed using Empower 3 software (Greenwood Village, CO, USA). For FTIR analysis, data were processed, and the graphical dependencies of wavenumbers (1/cm) on absorbance (–) for all gelatin samples were evaluated using Microsoft Office Excel 2016 (Denver, CO, USA). The MALDI Biotyper 3.0 identification database, Matrix-Assisted Laser Desorption/Ionization Time of Flight MS Biotyper (Bruker Daltonik GmbH, Bremen, Germany), evaluated the results of microbiological tests.

### 3.8. The Practical Relevance of the Work

The ideal solution to the growing demand for gelatin is offering an alternative raw material source, such as poultry collagen. Compared to earlier gelatin extraction research, this study is unique in using food-grade enzymes for targeted changes in collagen molecular structure before the extraction of gelatins. The implications of the studied chicken gelatin properties compared to traditional gelatins from porcine or bovine sources mainly consist of the use of chicken gelatins with no restrictions. The microbial purity of chicken gelatins makes them suitable for a wide range of advanced applications. The high antioxidant activity (more than 80% for the ABTS) of gelatins enables them to be used in cosmetics to produce antiaging creams and gels and to develop new pharmaceuticals. Gelatins with a higher molecular weight (M_w_ > 74 kDa) are suitable for gel- and film-forming purposes to produce soft and hard gelatin capsules to encapsulate drugs, oils, food additives, or vitamins. The film-forming properties of chicken gelatins are of interest for packaging applications, coatings for foods and agricultural chemicals, or medical dressings. The excellent gel-forming properties of gelatins predispose them for various food applications in meat and fish products (jellies, binders, aspics), confectionery (gums, marshmallows), desserts (puddings, spreads), and milk products (yogurts, fresh-cheese-based products).

## 4. Conclusions

This paper presents results on the properties of chicken gelatins at an advanced molecular level, which have not been previously studied. In addition to chains with molecular weights of less than 80 kDa, typical α- (80–160 kDa), β- (160–250 kDa), and γ- (250–375 kDa) chains were in all found chicken gelatin samples. The gelatin solutions showed an antioxidant activity of >69% towards DPPH and >80% towards ABTS; the antioxidant activity increased with increasing gelatin concentration. FTIR analysis confirmed all typical vibrational regions of Amides A and B, and Amides I, II, and III at characteristic wavenumbers. Microbiological analysis showed that no undesirable bacteria (*Salmonella*, *Listeria monocytogenes*, and *Escherichia coli*) were present in gelatins. Gelatin from chicken stomachs offers considerable potential for new advanced applications, such as in gels as a pharmaceutical carrier, and in products based on hemostatic agents. This new resource is a contribution to the circular economy by disposing slaughterhouse by-products and valorizing raw materials for gelatin production. Mild processing conditions are required for gelatin production from chicken stomachs collagen compared to bovine or porcine collagen processing. The treatment of purified chicken collagen requires a low amount of proteolytic enzyme, and gelatin is extracted at temperatures below 70 °C for a short time (45 min). Enzymatic treatment does not use strong acids or alkalis, allows for less water and raw material waste, reduces processing time, saves energy, and eliminates undesirable environmental aspects.

## Figures and Tables

**Table 1:** Values of scavenged free radicals for DPPH and ABTS in gelatin solutions at concentrations of 2, 4, 6, 8, and 10 mg/mL.

**DPPH**					
**Exp.** **No.**	**Gelatin Concentrations (mg/mL)**				
	**2**	**4**	**6**	**8**	**10**
1	72.16 ± 0.83 ^b,c,d,e^	76.28 ± 0.72 ^a,c,d,e^	82.04 ± 1.08 ^a,b,d^	84.79 ± 1.52 ^a,b,c,e^	87.44 ± 1.70 ^a,b,d^
2	70.92 ± 0.71 ^b,c,d,e^	72.43 ± 1.92 ^a,c,d,e^	80.45 ± 0.89 ^a,b,d^	82.18 ± 0.65 ^a,b,c,e^	86.79 ± 1.27 ^a,b,d^
3	71.46 ± 1.76 ^b,c,d,e^	77.70 ± 0.76 ^a,c,d,e^	82.08 ± 0.71 ^a,b,d^	82.41 ± 1.57 ^a,b,c,e^	86.44 ± 0.51 ^a,b,d^
4	71.57 ± 1.60 ^b,c,d,e^	76.62 ± 1.89 ^a,c,d,e^	87.83 ± 1.03 ^a,b,d^	84.88 ± 1.83 ^a,b,c,e^	87.09 ± 0.73 ^a,b,d^
5	69.48 ± 0.87 ^b,c,d,e^	75.59 ± 1.01 ^a,c,d,e^	77.12 ± 1.87 ^a,b,d^	82.42 ± 0.78 ^a,b,c,e^	84.80 ± 0.95 ^a,b,d^
6	68.94 ± 0.66 ^b,c,d,e^	73.60 ± 0.67 ^a,c,d,e^	77.91 ± 0.91 ^a,b,d^	83.90 ± 1.02 ^a,b,c,e^	86.93 ± 1.70 ^a,b,d^
7	70.76 ± 1.03 ^b,c,d,e^	76.55 ± 0.80 ^a,c,d,e^	81.72 ± 1.80 ^a,b,d^	83.71 ± 1.44 ^a,b,c,e^	87.01 ± 1.33 ^a,b,d^
8	71.94 ± 0.79 ^b,c,d,e^	75.90 ± 0.81 ^a,c,d,e^	79.25 ± 1.93 ^a,b,d^	84.28 ± 1.20 ^a,b,c,e^	86.92 ± 1.83 ^a,b,d^
9	70.42 ± 0.88 ^b,c,d,e^	74.33 ± 1.97 ^a,c,d,e^	77.27 ± 0.44 ^a,b,d^	84.43 ± 0.88 ^a,b,c,e^	86.87 ± 1.90 ^a,b,d^
$\bar{x}$ ± SD	70.85 ± 1.01	75.50 ± 1.14	80.63 ± 1.18	83.67 ± 1.21	86.70 ± 1.32
**ABTS**					
**Exp.** **No.**	**Gelatin Concentrations (mg/mL)**				
	**2**	**4**	**6**	**8**	**10**
1	84.02 ± 1.55 ^c,d,e^	86.72 ± 1.61 ^c,d,e^	91.32 ± 1.76 ^a,b,d,e^	93.22 ± 1.23 ^a,b,c,e^	96.51 ± 1.11 ^a,b,c,d^
2	83.21 ± 1.72 ^c,d,e^	85.32 ± 0.79 ^c,d,e^	87.82 ± 1.55 ^a,b,d,e^	92.77 ± 0.80 ^a,b,c,e^	94.87 ± 0.93 ^a,b,c,d^
3	79.66 ± 0.87 ^c,d,e^	84.93 ± 1.33 ^c,d,e^	88.04 ± 0.71 ^a,b,d,e^	93.48 ± 1.72 ^a,b,c,e^	94.69 ± 1.40 ^a,b,c,d^
4	84.01 ± 0.93 ^c,d,e^	87.70 ± 1.70 ^c,d,e^	91.43 ± 0.93 ^a,b,d,e^	93.08 ± 1.51 ^a,b,c,e^	96.07 ± 0.61 ^a,b,c,d^
5	79.59 ± 1.77 ^c,d,e^	87.45 ± 1.62 ^c,d,e^	89.93 ± 1.62 ^a,b,d,e^	90.66 ± 1.42 ^a,b,c,e^	95.72 ± 1.14 ^a,b,c,d^
6	82.40 ± 1.04 ^c,d,e^	83.71 ± 1.81 ^c,d,e^	90.61 ± 1.42 ^a,b,d,e^	90.40 ± 1.08 ^a,b,c,e^	95.27 ± 1.37 ^a,b,c,d^
7	83.55 ± 1.83 ^c,d,e^	88.02 ± 0.93 ^c,d,e^	89.94 ± 1.27 ^a,b,d,e^	92.61 ± 1.76 ^a,b,c,e^	94.85 ± 0.59 ^a,b,c,d^
8	82.37 ± 1.45 ^c,d,e^	87.66 ± 1.37 ^c,d,e^	90.04 ± 1.51 ^a,b,d,e^	93.28 ± 1.48 ^a,b,c,e^	94.52 ± 1.69 ^a,b,c,d^
9	79.94 ± 1.30 ^c,d,e^	85.59 ± 1.22 ^c,d,e^	88.40 ± 1.22 ^a,b,d,e^	91.69 ± 0.79 ^a,b,c,e^	93.77 ± 1.33
$\bar{x}$ ± SD	82.08 ± 1.38	86.43 ± 1.38	89.73 ± 1.33	92.35 ± 1.31	95.14 ± 1.13

The letters in superscripts in the columns indicate significant differences (*p* < 0.05) between tested gelatin concentrations (^a^ = 2, ^b^ = 4, ^c^ = 6, ^d^ = 8, and ^e^ = 10 mg/mL) under changing experimental conditions.

**Table 2:** Molecular weight values for individual gelatin samples, including PDI.

Exp. No.	M_p_ ^1^ (kDa)	M_w_ ^2^ (kDa)	M_n_ ^3^ (kDa)	PDI (-)
1	18.4	24.5	5.6	4.4
2	41.0	65.1	8.3	7.9
3	44.1	94.9	8.6	11.1
4	18.8	30.5	6.0	5.1
5	19.2	57.3	6.4	9.0
6	57.5	105.1	9.6	11.0
7	17.5	24.4	5.3	4.6
8	18.7	45.8	7.0	6.5
9	38.7	74.0	8.3	8.9

^1^ Molecular weight of the peak maxima, ^2^ weight average molecular weight, ^3^ number average molecular weight.

**Table 3:** Percentages of molecular weights in gelatin samples.

Exp. No.	M_L_ (%)	M_α_ (%)	M_β_ (%)	M_γ_ (%)	M_H_ (%)
1	76.2	11.7	8.3	3.8	0.0
2	64.7	10.1	8.2	6.1	10.9
3	60.3	9.3	6.7	6.7	17.0
4	67.8	9.5	7.4	8.4	6.9
5	62.2	9.6	7.9	6.9	13.4
6	59.9	9.3	6.5	7.4	16.9
7	64.6	10.4	7.0	7.9	10.1
8	63.4	9.5	7.5	6.7	12.9
9	58.3	10.4	7.9	6.5	16.9
$\bar{x}$ ± SD	65.0 ± 5.0	10.0 ± 1.0	7.0 ± 1.0	7.0 ± 1.0	12.0 ± 6.0

M_L_—the values of molecular weights 0–80 kDa, M_α_—the values of molecular weights 80–160 kDa (α-chains), M_β_—the values of molecular weights 160–250 kDa (β-chains), M_γ_—the values of molecular weights 250–375 kDa (γ-chains), M_H_—the values of molecular weights > 375 kDa.

**Table 4:** Results of FTIR peak regions for chicken gelatins, with reference regions.

Peak	Reference (1/cm) ^1^	Note	Exp. No.	Wavenumbers (1/cm)	Exp. No.	Wavenumbers (1/cm)	Exp. No.	Wavenumbers (1/cm)
Amide A	3440–3300	N–H stretching	1	3282	4	3289	7	3276
			2	3275	5	3272	8	3286
			3	3290	6	3277	9	3290
Amide B	3080–2899	CH_2_ asymmetrical stretch	1	2937	4	2938	7	2936
			2	2933	5	2932	8	2934
			3	2927	6	2929	9	2925
Amide I	1700–1600	C=O stretching	1	1640	4	1636	7	1641
			2	1644	5	1643	8	1645
			3	1639	6	1637	9	1644
Amide II	1580–1500	N–H bending	1	1515	4	1515	7	1519
			2	1517	5	1522	8	1525
			3	1510	6	1517	9	1525
Amide III	1350–1200	N–H bending and C–N stretching	1	1241	4	1236	7	1245
			2	1240	5	1240	8	1242
			3	1238	6	1238	9	1235

^1^ References [58,59,60,61,62].

**Table 5:** Microorganisms detected in gelatin samples, including the score value.

Exp. No.	*Organism*	Score Value	Exp. No.	*Organism*	Score Value
1	*Brevibacillus agri* *Bacillus cereus*	1997 ^c^ 1755 ^c^	6	*-*	-
2	*Bacillus cereus* *Bacillus flexus* *Brevibacillus agri*	2265 ^b^ 2093 ^b^ 1995 ^c^	7	*Bacillus cereus* *Acinetobacter radioresistens*	2239 ^b^ 1758 ^c^
3	*Acinetobacter radioresistens* *Acinetobacter baumannii*	2431 ^a^ 2004 ^b^	8	*Acinetobacter baumannii*	2359 ^a^
4	*Enterococcus faecium* *Bacillus flexus* *Brevibacillus agri*	2065 ^b^ 1835 ^c^ 1708 ^c^	9	*Acinetobacter baumannii* *Brevibacillus agri*	2121 ^b^ 2012 ^b^
5	*Acinetobacter baumannii* *Staphylococcus hominis* *Bacillus cereus*	2071 ^b^ 1801 ^c^ 2106 ^b^			

^a^ Highly probable species identification (2300–3000), ^b^ secure genus identification, probable species identification (2000–2299), ^c^ probable genus identification (1700–1999).

**Table 6:** Gelatin samples with the technological conditions of the extraction process [34].

Exp.	Factor A	Factor B	Exp.	Factor A	Factor B
No.	Enzyme (%)	Temperature (°C)	No.	Enzyme (%)	Temperature (°C)
1	0.10	55.0	6	0.15	70.0
2	0.10	62.5	7	0.20	55.0
3	0.10	70.0	8	0.20	62.5
4	0.15	55.0	9	0.20	70.0
5	0.15	62.5			

**Table 7:** Types of molecular weights and PDI, determined by GPC-RID analysis.

Molecular Weight Species	Unit	Note
M_p_	kDa	Molecular weight of the peak maxima
M_w_	kDa	Number average molecular weight
M_n_	kDa	Weight average molecular weight
PDI	-	Polydispersity index

**Table 8:** Characteristic FTIR peak vibrational regions for powdered gelatins [60].

Peak	Reference (1/cm)	Note
Amide A	3440–3300	N–H stretching
Amide B	3080–2899	CH_2_ asymmetrical stretch
Amide I	1700–1600	C=O stretching
Amide II	1580–1500	N–H bending
Amide III	1350–1200	N–H bending and C–N stretching

**Table 9:** Identification criteria for gelatin samples [18].

Score	Note
2300–3000	Highly probable species identification
2000–2299	Secure genus identification, probable species identification
1700–1999	Probable genus identification
<1700	Not reliable identification

## Data Availability

Data is contained within the article.
